# A measure of identifying influential waypoints in air route networks

**DOI:** 10.1371/journal.pone.0203388

**Published:** 2018-09-18

**Authors:** Guangjian Ren, Jinfu Zhu, Chaoyang Lu

**Affiliations:** Nanjing University of Aeronautics and Astronautics, Nanjing, China; Rutgers The State University of New Jersey, UNITED STATES

## Abstract

As the basic carrier of air flight operation, air route network (ARN) is of great significance to the smooth operation of flights. However, the waypoint is a core part of the route, so it is an important topic to identify influential waypoints in ARN. In this paper, a method to identify the influence of the node in ARN based on an improved entropy weight (IEW) method is proposed. Then, centrality measures including degree, closeness, betweenness and eigenvector as the multi-attribute of ARN in IEW application. IEW method is used to aggregate the multi-attribute to obtain the evaluation of the influence of each waypoint. To demonstrate the effectiveness of the IEW method, three real ARNs are selected to conduct several experiments with susceptible infected recovered (SIR) model. The results show the efficiency and practicability of the proposed method.

## Introduction

The airplane currently does not follow a smooth and optimized trajectory. Yet, they should follow a path on a predefined grid whose nodes are called navigation points or waypoints. Therefore, the air route network (ARN) as the basic operation carrier of air transportation, is of great significance to the smooth operation of flights. During the ARNs, route segments (edges) are connected by a series of air route waypoints (nodes) and the waypoints include the navigation stations, crossing points and reporting points.

With the waypoints’ help, pilots can have exact positions from the ground navaids to keep on the right track. In addition, the waypoint is also where the flight is concentrated. For example, according to the statistics of the Civil Aviation Administration of China (2017), the busiest waypoint is ZHO (A waypoint). Its flight volume reached more than 1,700 vehicles daily. Besides, the number of flights in the top-10 busy waypoints exceeded 850 vehicles daily.

Key waypoints affects the efficiency of aviation operations, moreover, they have a significant impact on the safety and reliability of aviation operations. Therefore, identification of key waypoints has important influence on the safety and reliability of aviation operations and at the same time contributes to the reduction of airborne delays.

ARNs can be simplified as the undirected networks and analyzed within the framework of complex network theory. Identifying influential nodes is a fundamental and practical topic in the research of complex networks. Opsahl et al.[1] use generalizing degree and shortest paths to study node centrality in weighted networks and the result has certain accuracy, but it mainly focus on local characteristics, lack of comprehensiveness. A semi-local centrality measure as a tradeoff between the low-relevant degree centrality and other time-consuming measures (betweenness or closeness) is proposed to identify influential nodes in [2]. Chen [3] design three metrics to assess system homogeneity, diffusion speed, and diffusion scale, and investigate their performance over complex systems, results show that they are locally optimal but not necessarily globally optimal. Community structure is also used to help identify influential nodes in [4], but the result is affected by the size of the communities. A new efficiency centrality (EffC) to rank the spreaders in the whole network is proposed in [5] to study influential nodes. Besides, both the k-shells theory [6] and the message-passing approach [7] are proposed to identify influential spreaders in complex networks. Chen et al.[8] discuss influential nodes in large-scale directed networks by proposing a local ranking algorithm named ClusterRank, and the theory is effective but mainly based on local information, lacking global characteristics. Furthermore, hybrid degree centrality (HC) are used to analyze the importance of nodes in weighted networks [9], and this theory improves the stability of the results to some extent, but ignores the local structure of neighbors. In short, the above theory solves the issue of identifying influential nodes at a different angle, and has certain accuracy and applicability. However, most of them are singular and lack comprehensive characteristics. For example, degree is a local centrality theory, and the betweenness and closeness are the global centrality ones. Therefore, the theories lack of comprehensiveness and applicability to some extent.

In addition, recently, the comprehensive centrality theories have also been enriched. The comprehensive centrality measure based on the Dempster-Shafer (DS) evidence theory is proposed in [10] and [11] to identify influential nodes. The results show that the DS theory is effective, however, the DS centrality is more focused on unweighted networks, lacking universality. Li and Deng[12] modify TOPSIS centrality method with the relative entropy and the effectiveness of the proposed method is demonstrated, but it lacks applicability more or less because it is sometimes difficult to select the appropriate centrality measures in some specific networks. Besides, an algorithm with weighted formal concept analysis (WFCA) is discussed in [13] and experiments illustrate that the WFCA can rank nodes effectively. A measure named community-based mediator (CbM) is proposed in [14], and the CbM describes how the node is essential to connect two or more than two communities of the network. The simulation shows that the theory performs well. What’s more, in [15], the Analytic Hierarchy Process (AHP) theory is used to identify influential nodes in the network and the efficiency and practicability of the method is proved by real networks. Nevertheless, the AHP has many subjective qualitative components. Although it reflects the subjective well, but it lacks scientificity and stability and the result is not easy to be convinced to a certain extent. Tian and Deng[16] put forward a new measure to identify the influential nodes based on information entropy and the theory includes not only local but global structure information. Results demonstrate that the proposed method can successfully identify the influential nodes in networks. Similarly, in this paper, a centrality measure based on entropy weight method is proposed. The proposed measure is an objective weighting method and it extracts information from the sample, namely the original data. Moreover, the deviation of the weight obtained by the method is smaller than that of the subjective weighting method (E.g. AHP) and the proposed method can better reflect the true importance of each evaluation index.

On the other hand, the infrastructure of aviation industry is analyzed by the universal use of complex network theory. The network structure and nodal centrality of individual cities in the air transport network of China are analyzed by centrality metrics in [17]. A critical infrastructure with an enormous impact on local, national, and international economies is discussed in the worldwide air transportation network [18]. In [19], a new network model is proposed, which takes the airport as a node and uses the airport traffic flow as the edge to investigate the network properties, and identifies the relevance of key airports in the network. Based on the situation, a model of multi-objective optimization for crossing waypoints location problem (CWLP) is built in [20], and the model is judged by the two factors of total airline cost and total flight conflict coefficient and proceed from the economic and safe aspects of air route network. Sun et al.[21] analyze the air navigation route system of fifteen different countries by using the following five metrics: degree, distance strength, weighted betweenness centrality, weighted closeness centrality, and edge length distribution. In [22], Cai et al. study the topology structure of the Chinese air route network (CARN) with complex network theory.

Most of the above analyses are based on the airport networks, that is, the airports are the nodes. If there are flights between airports, then add an edge. All of them can be used as a theoretical guide to my work, but they all lack research on air route networks (ARNs), especially the identification of key waypoints (nodes) where the nodes include the navigation stations, crossing points and reporting points. Based on the research above, in this paper, we analyze the structure characteristics of China's three typical regional (Beijing, Shanghai and Guangzhou) route networks and then use a proposed improved entropy weight centrality (IEWC) as well as other measures to study the influence of waypoints in route networks. The IEWC is a kind of objective weight method and it uses the information entropy to calculate the entropy weight of each index, and then modifies the weight of each index by entropy weight. Therefore, the weight of the index is objective and accurate, which can effectively avoid the influence of the subjective judgment (E.g. AHP) error on the weight analysis. In addition, the IEWC method can make full use of attribute information, combine the local (degree) and global (betweenness or closeness) characteristics of the network, and also consider the structure features of the neighbor nodes, which can better identify the influential nodes. As a result, the proposed measure is proved to be stable and effective by SIR model.

The structure of this paper is as follows. Some centrality measures for nodes are introduced in section 2 and the improved entropy weight centrality (IEWC) measure is proposed in section 3. Then in section 4, three real air route network examples are illustrated to show the efficiency and practicability of the proposed method, the SIR model and Kendall’s tau coefficient are used to evaluate the performance. Finally, section 5 concludes this paper and future work.

## Centrality measures for nodes

In every system in nature, there are always one or more elements that occupy a very important position. If you remove them, the system will be greatly affected in terms of structure, stability, and even survival. Therefore, the importance of each node in a complex network abstracted by a complex system is different. In a variety of complex networks, using a quantitative analysis method to find which node is the most important in a large-scale network, or the importance of a node relative to one or more other nodes, is a fundamental issue in the study of complex networks. The measure of the importance of nodes is the node centrality, which is used to quantitatively indicate that some nodes in the network are more important or more central than other nodes. This indicator is used to determine the relationship between the location of an individual in the network and its influence or appeal in the group.

Complex network centrality analysis focuses on the importance evaluation measures of the nodes, and on this basis reflects the degree of centralization of the entire network. According to the theory of complex networks, there are several measures for characterizing the centrality of a node in a graph, which are degree, closeness, betweenness and eigenvector centrality.

For a simple network *G* = (*V*,*E*) with *N*(= |*V*|) nodes and *M*(= |*E*|) edges, it can be described by an adjacency matrix *A* = {*a*_*ij*_}, where *a*_*ij*_ = 1 if node *v*_*i*_ is connected with node *v*_*j*_, and *a*_*ij*_ = 0 otherwise.

Degree of node *v*_*i*_ is defined as the number of edges connected to node *v*_*i*_.The degree centrality (DC) of node *v*_*i*_, denoted by *D*_*E*_(*v*_*i*_) is [23, 24]

$$D_{E}(v_{i})=\sum_{i=1}^{N}a_{ij}$$ (1)

Betweenness of node *v*_*i*_ is defined as the fraction of shortest paths between node pairs that pass through the node of interest, which reflects the role and influence of the node in the entire network [25, 26]. The betweenness centrality (BC) of node *v*_*i*_, denoted by *B*(*v*_*i*_) is expressed as follow

$$B(v_{i})=\sum_{s\neq i\neq t\in V}\frac{n_{st}(v_{i})}{n_{st}}$$ (2)

where *n*_*st*_ is the number of shortest paths between nodes *v*_*s*_ and *v*_*t*_, and *n*_*st*_(*v*_*i*_) denotes the number of shortest paths between *v*_*s*_ and *v*_*t*_ which pass through node *v*_*i*_.

Closeness centrality (CC) [27, 28], *C*_*C*_(*v*_*i*_) of node *v*_*i*_ is defined as the reciprocal of the sum of geodesic distances to all other nodes of *V*, and calculated by the following formula

$$C_{C}(v_{i})={\left[\sum_{j=1,j\neq i}d_{ij}\right]}^{-1}$$ (3)

where *d*_*ij*_ is the geodesic distance between *v*_*i*_ and *v*_*j*_.

Dangalchev (2006) modified the definition to a general form, called residual closeness in [29]. Residual closeness is able to reflect the effects of node removal even if this removal does not result in disconnected components, and it is expressed as

$$C_{R}(v_{i})=\sum_{j=1,j\neq i}2^{-d_{ij}}$$ (4)

*A* represents the adjacency matrix of graph (and *λ*_1_,*λ*_2_,⋯,*λ*_*N*_ are the eigenvalues of matrix *A*. The maximum eigenvalue of matrix *A* is *λ*_max_, and its corresponding eigenvector is *e* = [e_1_,e_2_,⋯e_*N*_]^*T*^. The formula is obtained as follow

$$\lambda_{\max}e_{i}=\sum_{j=1}^{N}a_{ij}e_{j}$$ (5)

The eigenvector centrality (EC) of node *v*_*i*_, denoted as *C*_*E*_(*v*_*i*_) is computed by [30, 31]

$$C_{E}(v_{i})=\lambda_{\max}^{-1}\sum_{j=1}^{N}a_{ij}e_{j}$$ (6)

## Improved entropy weight method

### Entropy weight centrality

Entropy is a physics concept that can be used as a measure of the degree of confusion within the system. In general, the larger the system entropy, the more microscopic states the system has in its macro state, and the more chaotic and unordered it is. On the contrary, the less the microscopic state of the system, the more uniform the internal state of the system, the more ordered, the smaller the entropy.

When there are *h* items to be evaluated and the evaluation indexes are *g*, forming original evaluation matrix *O* = (*o*_*ij*_)_*h*×*g*_ (*i* = *1*,*2*,⋯,*h*; *j* = *1*,*2*,⋯,*g*), then for each index *o*_*j*_, the Information entropy is

$$e_{j}=-\sum_{i=1}^{h}p_{ij}\cdot \ln\;p_{ij},\;\mathrm{where},\;p_{ij}=o_{ij}/\sum_{i=1}^{h}o_{ij}$$ (7)

If the entropy *e*_*j*_ of an index is small, it shows that the larger variability value of the index, the more information is provided, the bigger effect of this index in the comprehensive evaluation, and the greater the weight. Otherwise, if the entropy *e*_*j*_ of an index is big, it shows that the smaller variability value of the index, the less information is provided, the smaller effect of this index in the comprehensive evaluation, and the smaller the weight.

The entropy weight method is used to quantify the information for each item (network nodes) to be evaluated and gives the weight of each index (such as degree, betweenness and closeness, etc.) to simplify the evaluation process. Objective weight of each index can be determined by using entropy weight method firstly, and then the subjective weight can be revised by objective weight. The theory analysis is described as follows.

To express the IEW model, several new symbols and their definitions are introduced in Table 1.

**Table 1:** New symbols and their definitions.

Symbols	Definitions
*x*_*ij*_	The elements of index matrix, it means the value of the *ith* node is measured by *jth* centrality measure which *i* ∈ (1,2,3,⋯*n*), *j* ∈ (1,2,3,⋯*k*).
*n*	The number of network nodes to be evaluated.
*k*	The types of centrality measures.
*Y*	The normalized form of the matrix *X*.
*y*_*ij*_	The elements of matrix *Y*.
*μ*	The adjustment coefficient, which *μ* = 1/log(*n*).
*H*(*Z*)	Entropy value of the element *y*_*ij*_, which *i* ∈ (1,2,3,⋯*n*), *j* ∈ (1,2,3,⋯*k*).
*w*_*j*_	Weight value of the *jth* type of centrality measure, which $\sum_{j=1}^{k}w_{j}=1$.
*I*	The synthetic entropy weight matrix.

Firstly, in this section, the evaluation indicators are determined and the initial decision matrix (*x*_*ij*_)_*n*×*k*_ is obtained as follow

$$X={\left[\begin{array}{cccc} x_{11} & x_{12} & \cdots & x_{1k}\\ x_{21} & x_{22} & \cdots & x_{2k}\\ \vdots & \vdots & \vdots & \vdots \\ x_{n1} & x_{n2} & \cdots & x_{nk} \end{array}\right]}_{n\times k}$$ (8)

where *x*_*ij*_ is the *jth* index value of the *ith* network node, which *i* = 1,2,3,⋯,*n* and *j* = 1,2,3,⋯,*k*.

To make indexes of matrix *X* being dimensionless, and then we get the nondimensionalized factor, denoted by *y*_*ij*_ is [32]

$$y_{ij}=\frac{x_{ij}}{\sum_{i=1}^{n}X_{ij}},(j=1,2,3,\cdots ,k)$$ (9)

where *x*_*ij*_ is the element of matrix *X*.

The normalized decision matrix *Y* is

$$Y={\left[\begin{array}{cccc} y_{11} & y_{12} & \cdots & y_{1k}\\ y_{21} & y_{22} & \cdots & y_{2k}\\ \vdots & \vdots & \vdots & \vdots \\ y_{n1} & y_{n2} & \cdots & y_{nk} \end{array}\right]}_{n\times k}$$ (10)

Before the entropy weight of the index is determined, the following formula is defined.

$$H(Z_{j})=-\mu \sum_{i=1}^{n}y_{ij}\log(y_{ij}),j=1,2,3,\cdots ,k$$ (11)

where *μ* is the adjustment coefficient, that is, *μ* = 1/log (*n*).

The entropy weight of index, denoted by *w*_*j*_ is computed by

$$w_{j}=\frac{1-H\left(Z_{j}\right)}{k-\sum_{j=1}^{k}H\left(Z_{j}\right)},j=1,2,3,\cdots ,k$$ (12)

where 0 ≤ *w*_*j*_ ≤ 1 and $\sum_{j}^{k}w_{j}=1$.

The entropy weight has the following characteristics.

1. The entropy weight is not determined by human subjectivity and it is determined objectively by information entropy, avoiding the randomness of human factors.
2. When the value of each node on index *j* (centrality measure) is nearly equal, the entropy value is also close to the maximum value 1 and the entropy weight is close to 0. This means that the index can not provide sufficient information to the decision maker and the index can be ignored.
3. On the other hand, when the value of each node on index *j* differs greatly, the entropy is small and the entropy weight is large. This shows that the index provides more information for decision makers and this index should be focused on.
4. The entropy weight is not an important coefficient in the actual sense of the index, but is a relatively fierce degree in the competitive sense of each index under given networks and indexes.
5. From the perspective of information theory, entropy weight represents how much useful information an index provides.

At last, the entropy weight matrix *W* of indices is described as follow

$$W={\left[w_{1},w_{2},w_{3},\cdots ,w_{k}\right]}^{T}$$ (13)

The improved entropy weight centrality (IEWC) of node *v*, denoted by *I*_*c*_(*v*) is obtained by multiplying *Y* and *W*.

$$I_{C}(v)=YW$$ (14)

where *I*_*c*_(*v*) = [*I*_*c*_(*v*_1_),*I*_*c*_(*v*_2_),*I*_*c*_(*v*_3_),⋯,*I*_*c*_(*v*_*n*_)]^*T*^.

### Example demonstration

**Fig 1:**
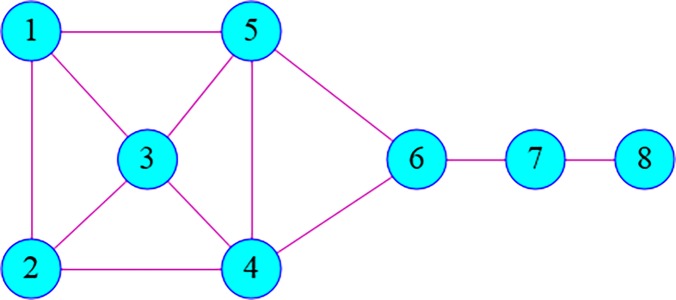
A simple network.

In this section, a simple network with 8 nodes is built (see Fig 1). The application of the proposed method is explained by calculating DC, BC, CC and EC of the nodes in the graph by turns, and analyzing the IEWC of the network.

As shown in Fig 1, the network is symmetrical. Intuitively, the centrality of node 1 is equal to node 2 and the same with nodes 4 and 5, respectively. Based on the proposed algorithm above, the initial decision matrix *X* of the network in Fig 1 is as follow

$$X=\left[\begin{array}{ccccc} nodes & DC & BC & CC & EC\\ node1 & 3 & 0.01587 & 0.5 & 0.14735\\ node2 & 3 & 0.01587 & 0.5 & 0.14735\\ node3 & 4 & 0.03175 & 0.5385 & 0.18737\\ node4 & 4 & 0.23016 & 0.6364 & 0.17973\\ node5 & 4 & 0.23016 & 0.6364 & 0.17973\\ node6 & 3 & 0.47619 & 0.6364 & 0.11307\\ node7 & 2 & 0.28571 & 0.4667 & 0.03528\\ node8 & 1 & 0 & 0.3333 & 0.01011 \end{array}\right]$$ (15)

The matrix *X* is normalized by Eq (8), then we can get the matrix *Y*

$$Y=\left[\begin{array}{ccccc} nodes & DC & BC & CC & EC\\ node1 & 0.42857 & 0.01587 & 0.5 & 0.14735\\ node2 & 0.42857 & 0.01587 & 0.5 & 0.14735\\ node3 & 0.57143 & 0.03175 & 0.5385 & 0.18737\\ node4 & 0.57143 & 0.23016 & 0.6364 & 0.17973\\ node5 & 0.57143 & 0.23016 & 0.6364 & 0.17973\\ node6 & 0.42857 & 0.47619 & 0.6364 & 0.11307\\ node7 & 0.28571 & 0.28571 & 0.4667 & 0.03528\\ node8 & 0.14286 & 0 & 0.3333 & 0.01011 \end{array}\right]$$ (16)

According to Eq (11), the entropy weight matrix *W* of indices is

$$W={\left[\begin{array}{cccc} 0.3191 & 0.1662 & 0.3084 & 0.2063 \end{array}\right]}^{T}$$ (17)

On the basis of the Eq (13) above, the IEWC of the graph in Fig 1 is shown in Table 2

**Table 2:** IEWC of the nodes in Fig 1.

Fig 1	Node 1	Node 2	Node 3	Node 4	Node 5	Node 6	Node7	Node8
IEWC	0.3240	0.3240	0.3923	0.4539	0.4539	0.4355	0.2899	0.1505
Rank	node 4 = node 5 > node 6 > node 3 > node 1 = node 2 > node 7 > node 8							

In Table 2 node4 and node 5 are ranked as the most influential node in the IEWC method and this result is evident in Fig 1. In addition, it can be seen from Fig 1 that nodes1 and 2 are symmetrical so, the sorting position is equal to each other. Although the degree of node3 is greater than node 6, it is obvious that the position of 6 is more important. So the rank of node 6 is relatively high. Simultaneously, the centrality of node7 higher than 8. The above shows that the IEWC model can better reflect the importance of nodes to some extent. Thus, the objectivity, accuracy, and applicability of the proposed measure for nodes are demonstrated by the example.

## Experimental discussion

### Air route network data

**Fig 2:**
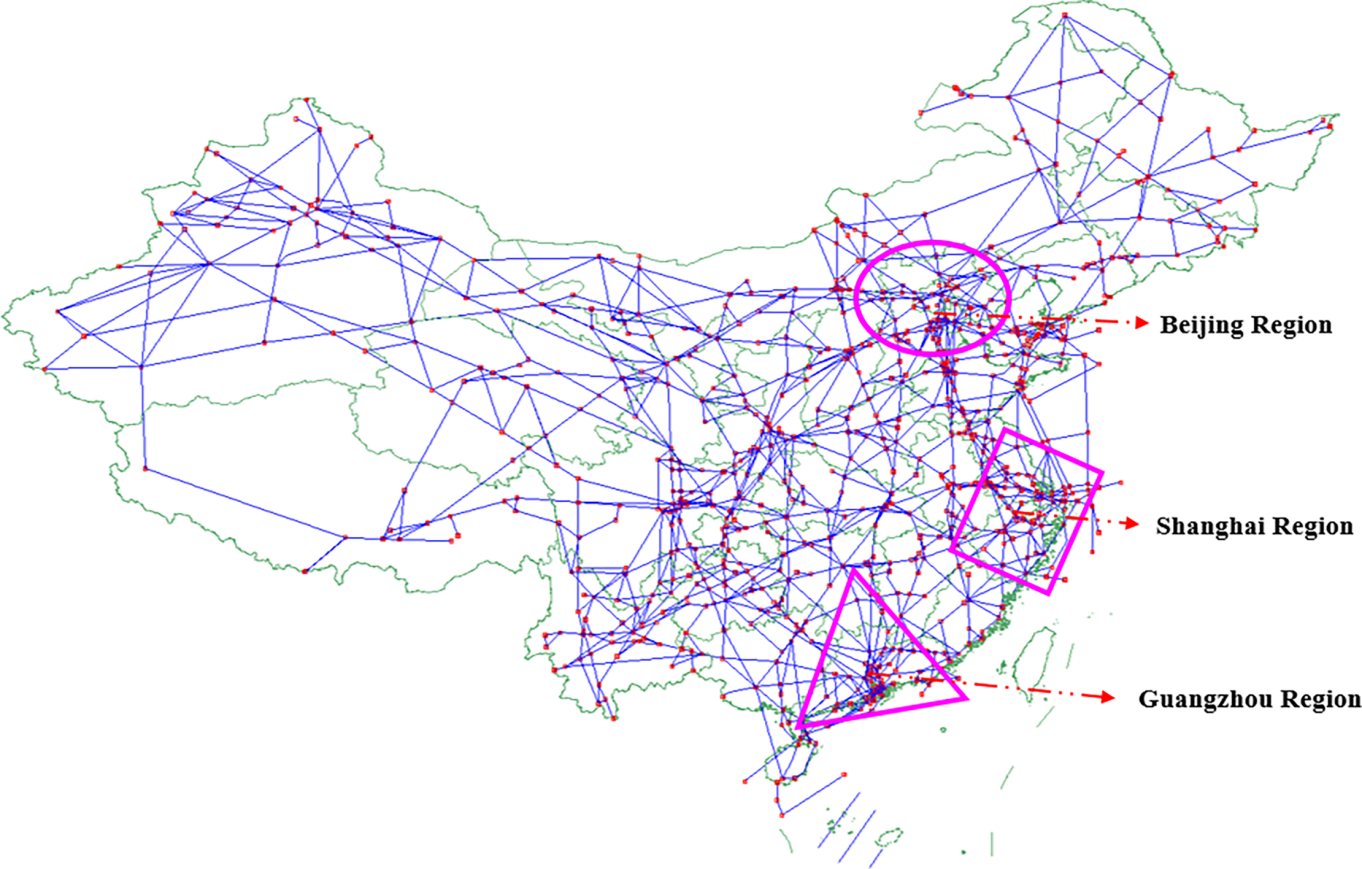
Chinese air route network.

In this section, the Chinese air route network (CARN) provided by the Air Traffic Management Bureau (ATMB) of China is selected as experimental data to verify the validity of the proposed model. The topology layout of CARN is shown in Fig 2.

**Fig 3:**
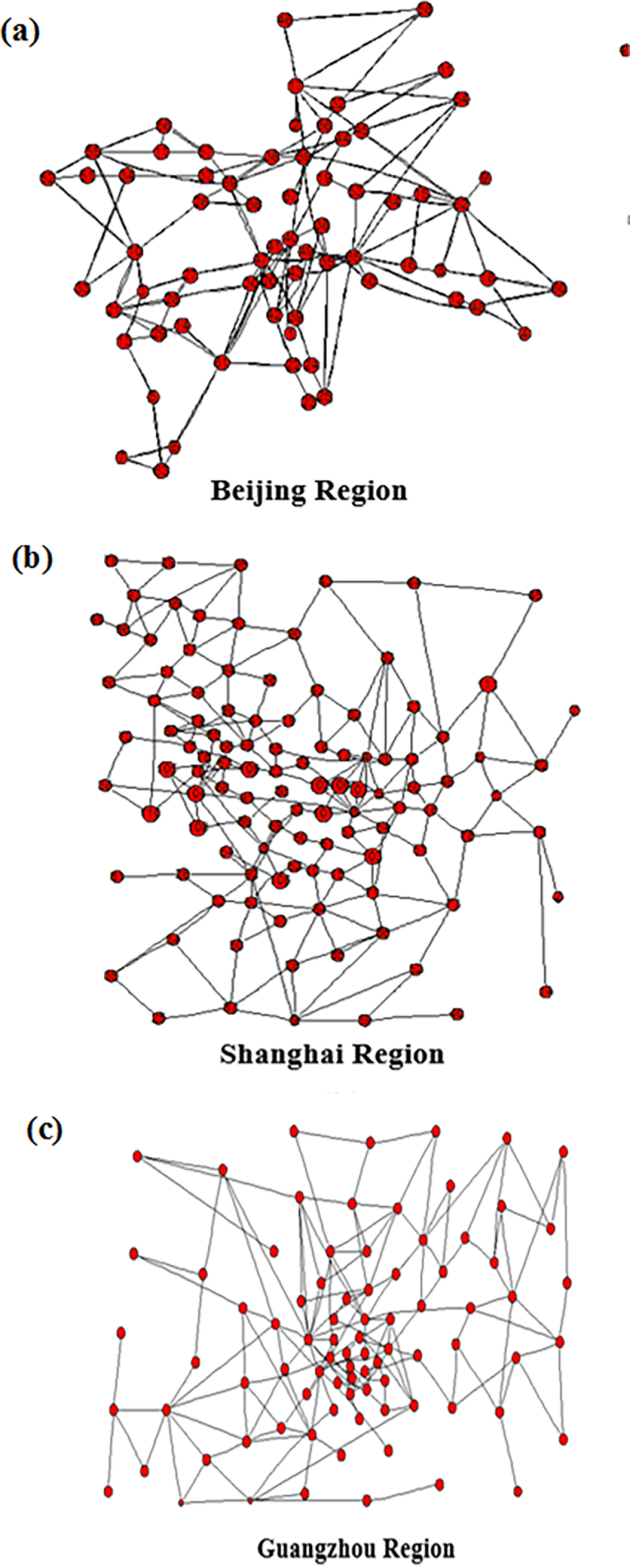
Top 3 subnets of CARN.

As depicted in Fig 2, the blue solid line represents the air route segments and they are connected to a series of air route waypoints (red nodes in Fig 2) where the waypoints (that is, nodes) include the airport navigation stations (not the airports), navigation points, crossing points and reporting points. Therefore, ARN can be simplified as an undirected complex network. According to the daily flight count statistics of the Civil Aviation Administration of China in 2017, the three busiest airspace regions in China are Beijing, Shanghai and Guangzhou regions triangle respectively (As shown in Fig 2, Beijing, Shanghai and Guangzhou regions are marked by pink ellipse, rectangle and triangle respectively.). In order to satisfy the need of the experiment, the detailed topology of top-3 subnets of CARN is shown in Fig 3.

Fig 3(A), 3(B) and 3(C) give a detailed representation of Beijing, Shanghai and Guangzhou air route networks respectively. By analyzing the network structure of each region in Fig 3, we can get the basic topological features of the three real air route networks (see Table 3).

**Table 3:** Basic topological features of the top-3 subnets of CARN.

Networks	Nodes	Edges	Average degree	Clustering coefficients	Assortativity coefficients
Beijing	69	254	3.6812	0.1722	-0.058
Shanghai	111	220	3.9459	0.2202	0.0217
Guangzhou	84	158	3.7738	0.1682	-0.024

Table 3 makes a simple comparison of the basic characteristics of the top-3 air route network. The average degree of the three networks is about 4, indicating that on average, the waypoint contains four route segments. Because of the similar clustering coefficients, the clustering effect of the air route networks is close to each other. Assortativity coefficients of the networks Beijing and Guangzhou are negative, which means that large-degree nodes are more likely to link the small-degree nodes to some degree. However, the parameter of Shanghai air route network is positive, that is, the large-degree nodes tend to connect large-degree ones.

### Overview of SIR model

The SIR model is one of the most classic models in the epidemic model and can be used to identify the influential nodes in complex networks. According to [33], SIR model has three states: (a) Susceptible, denoted by *S*(*t*) are generally the healthy individuals, but can be infected by viruses; (b) Infected *I*(*t*) is the individuals who have been infected with the virus and have the ability to infect other healthy individuals;(c) Recovered *R*(*t*) is the individuals who has never had the ability to infect other healthy individuals and is not infected by other infected individuals. Initially, let a test node infect, then each step will randomly infected susceptible neighbors, the probability of infection is *γ*. After a long period of time, due to the lack of susceptible individuals, the infected nodes gradually decreased. When no node is infected, the infection process ends. The total number of infected nodes and recovered nodes represented by time *t*, denoted by *F*(*t*), may be considered as an index for evaluating the influence of the tested node at time *t*. As time *t* continues to increase, *F*(*t*) increases and eventually remains stable. Where *t*_*d*_ indicates the terminal time when there is no infected node. Therefore, the final coverage of the node *i*, denoted by *F*_*i*_(*t*_*d*_), is used to represent the true propagation capabilities of *i*, where node *i* is set to initial infection. The influence of the node *i* is related to the value of *F*_*i*_(*t*_*d*_). The higher the value of *F*_*i*_(*t*_*d*_), the more impact the node has.

### Kendall’s tau coefficient

In order to evaluate the performance of different identifying influential nodes algorithms, Kendall’s tau coefficient, denoted by *τ* is introduced to measure the influence of node spreading effects on the correlation of the above five methods. Kendall’s tau coefficient is a statistic used to measure the correlation of two random variables.

Suppose two random variables are *X* and *Y* (also can be seen as two sets). Their number of elements is *N*, and the *i*th (1≤*i*≤*N*) values taken by the two random variables are denoted by *X*_*i*_ and *Y*_*i*_ respectively. The corresponding elements in *X* and *Y* form an element pair set *XY*, which contains elements (X_*i*_,Y_*i*_) (1≤*i*≤*N*). When *X*_*i*_ > *X*_*j*_ and *Y*_*i*_ > *Y*_*j*_, or *X*_*i*_ < *X*_*j*_ and *Y*_*i*_ < *Y*_*j*_, these two elements are considered to be consistent. When *X*_*i*_ > *X*_*j*_ and *Y*_*i*_ < *Y*_*j*_, or *X*_*i*_ < *X*_*j*_ and *Y*_*i*_ > *Y*_*j*_, these two elements are considered inconsistent. When *X*_*i*_ = *X*_*j*_ and *Y*_*i*_ = *Y*_*j*_, these two elements are neither concordant nor discordant. Kendall’s tau coefficient is defined as follow [34, 35]

$$\tau =\frac{N_{c}-N_{d}}{0.5n(n-1)},(-1\leq \tau \leq 1)$$ 18

Where *N*_*c*_ and *N*_*d*_ are the number of concordant and discordant pairs, respectively. The higher the *τ* value, the more accurate the list of rankings the method can generate.

### Effectiveness

In this section, we use the above theories in sections 2and 3 to obtain the top-10 waypoints of the three air route networks and compare the results. The SIR model mentioned in section 4.2 is also used to identify the influential nodes and the results are obtained by over 100 independent runs. Lists of top-10 influential waypoints for the three real networks are shown in Table 4.

**Table 4:** The top10 waypoints of the three air route networks based on the spreading ability *F*(*t*) (*t* = 10 and 100 implementations) and five centrality measures mentioned above.

Top 10	Centrality measures					
Beijing	DC	BC	CC	EC	Proposed method	*F*(t)
1	65	59	59	65	65	65
2	62	69	47	62	47	47
3	40	65	40	59	63	59
4	17	47	69	35	59	40
5	47	40	65	47	10	63
6	59	62	62	40	29	69
7	61	61	36	53	62	17
8	69	17	61	54	32	35
9	10	36	35	43	17	32
10	35	10	41	69	40	55
Shanghai						
1	8	104	104	8	8	8
2	4	92	8	5	104	4
3	84	8	91	4	4	5
4	92	73	92	100	41	92
5	104	4	4	11	92	104
6	3	91	65	12	20	84
7	5	9	3	102	11	3
8	9	65	66	107	3	11
9	11	3	11	20	35	63
10	12	11	69	54	84	20
Guangzhou						
1	59	59	59	59	59	59
2	64	51	51	64	6	64
3	6	66	64	51	51	51
4	51	64	66	57	40	66
5	57	42	46	46	47	57
6	66	6	32	35	64	40
7	1	1	34	61	57	6
8	40	46	57	32	39	47
9	47	39	55	55	16	46
10	30	40	61	38	32	32

It can be seen from Table 4 that in Beijing, the proposed IEWC, DC and *F*(*t*)have the same seven members in the top-10 lists, and especially the top-2 waypoints of the proposed method and *F*(*t*) are the same; while, the same number of the top-10 nodes among BC, CC, EC and *F*(*t*) is six. Therefore, the proposed IEWC can reflect the importance of nodes in Beijing networks better. In Shanghai, the same numbers in the top-10 lists among the proposed IEWC, DC and *F*(*t*) are eight; the proposed IEWC and DC have the same seven members in the top-10 lists; BC, CC and *F*(*t*) have the same number in the top-10 lists, but the same number of top-10 nodes between EC and *F*(*t*) is five. In Guangzhou, the result for the proposed method and DC is similar, because the same numbers in the top-10 lists between *F*(*t*) and the proposed IEWC or DC is eight, simultaneously, the quantity between the proposed IEWC and DC is seven. Both BC and CC have seven nodes with what *F*(*t*) ranked, but EC performs a little bad with six same nodes. Obviously, node 59 is the most influential one in Guangzhou. Overall, it can be found that the proposed method performs well in identify the influence of waypoints among the real air route networks.

**Fig 4:**
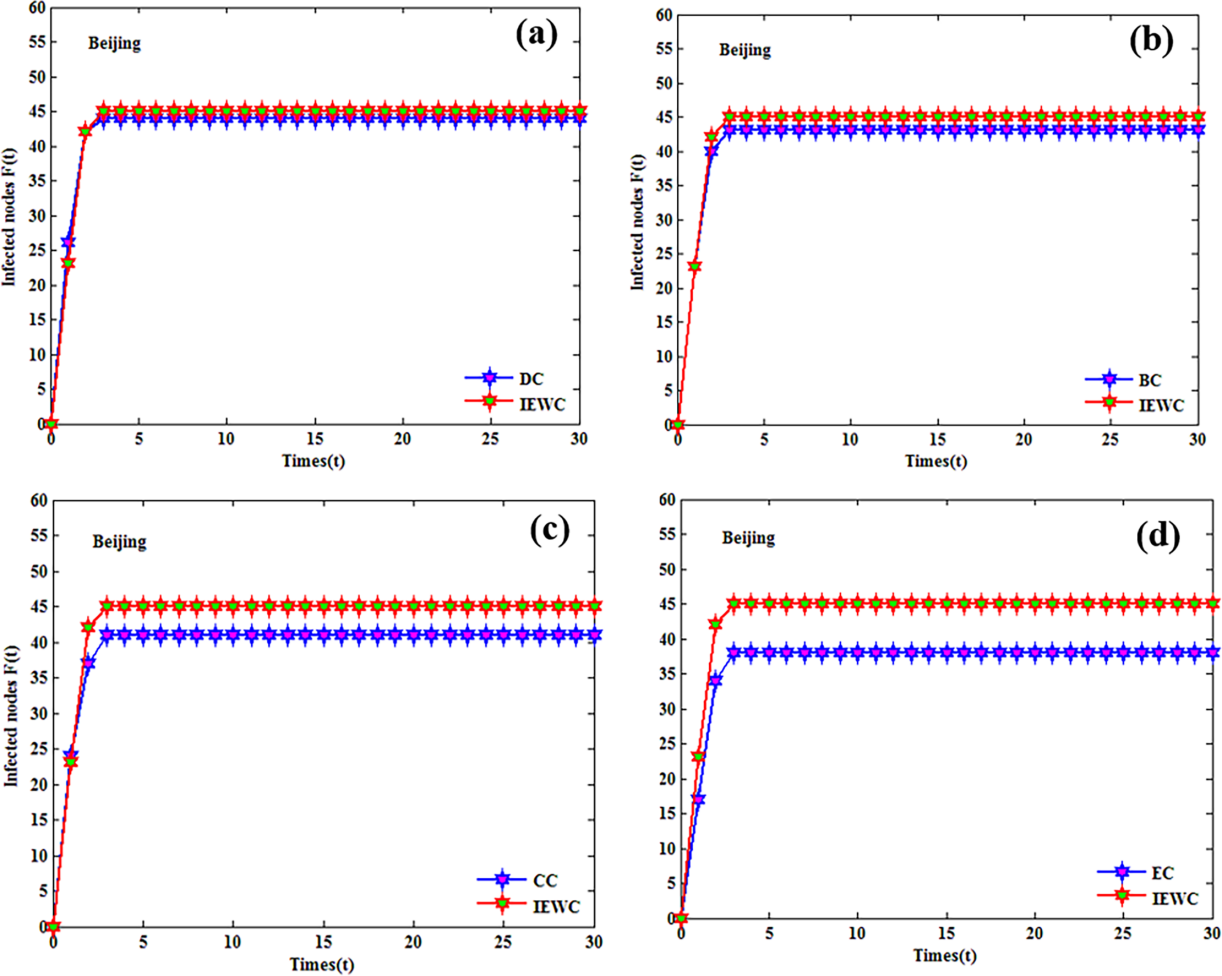
The cumulative number of infected nodes as a function of time in Beijing with the initially infected nodes that ranked in the top-10 lists by the proposed method or others. (a), (b), (c), (d).

**Fig 5:**
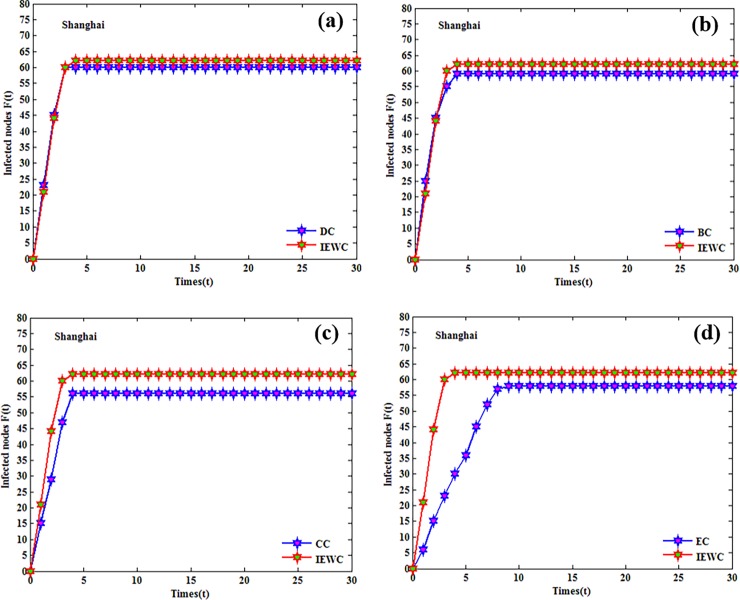
The cumulative number of infected nodes as a function of time in Shanghai with the initially infected nodes that ranked in the top-10 lists by the proposed method or others. (a), (b), (c), (d).

**Fig 6:**
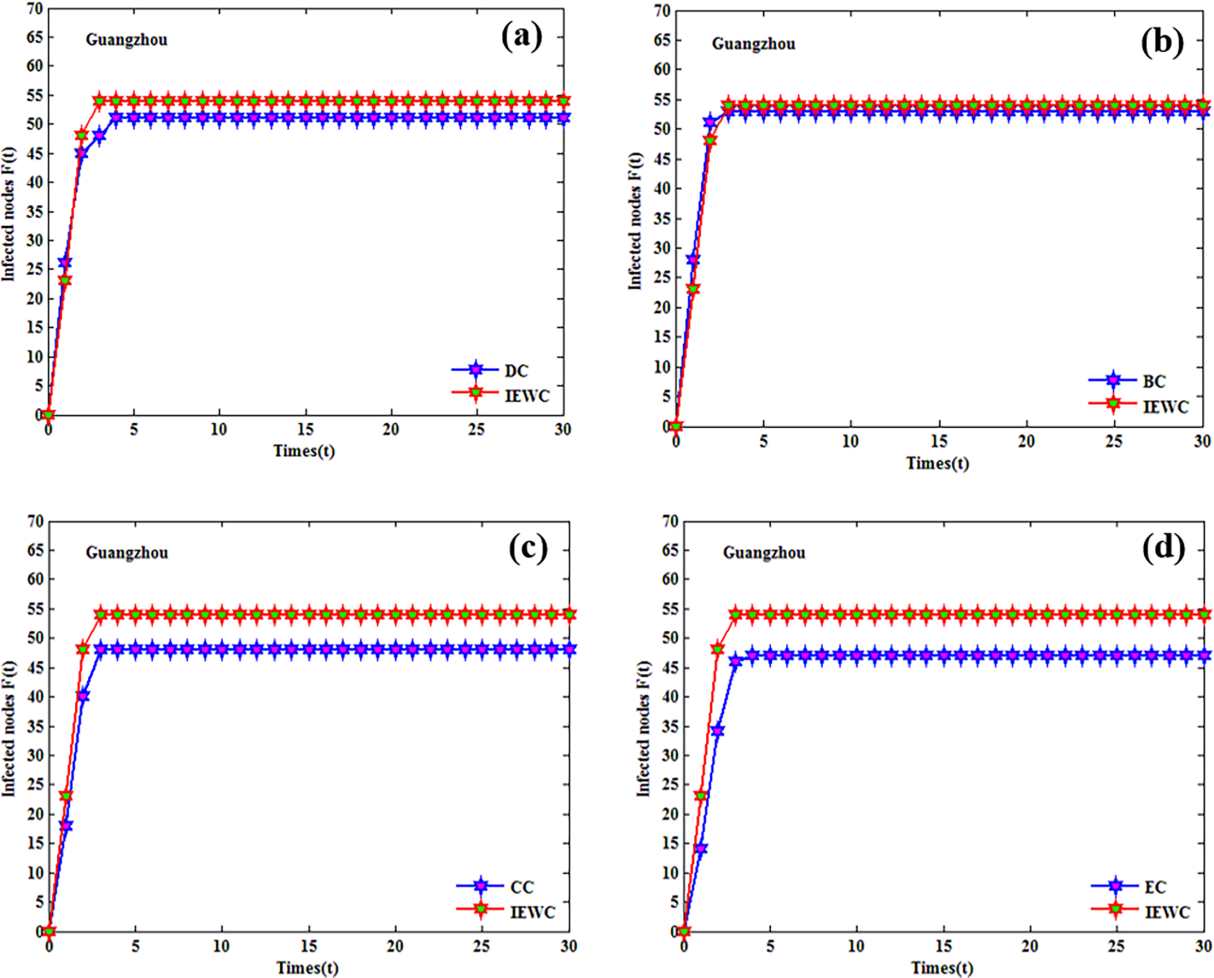
The cumulative number of infected nodes as a function of time in Guangzhou with the initially infected nodes that ranked in the top-10 lists by the proposed method or others. (a), (b), (c), (d).

The influence of the nodes that either appear in the top-10 list by the proposed method or others is compared with some simulations (over 100 runs). The cumulative infected nodes for these three air route networks are shown in Figs 4, 5 and 6.

In Beijing, the result for the proposed IEWC and DC is similar, because the lines of the proposed IEWC and DC almost overlap as shown in Fig 4(A). From Fig 4(B), we can find that the average number of infected nodes by the proposed IEWC is slightly higher than BC at each step. It is obvious that the proposed IEWC performs better than CC and EC, as presented in Fig 4(C) and 4(D). In Shanghai, both DC and BC have similar performance with the number of infected nodes is slightly smaller than the proposed method (see Fig 5(A) and 5(B)). As depicted in Fig 5(C), the proposed IEWC outperforms CC. At the same time, the performance of BC as shown in Fig 5(D) is not as good as the proposed IEWC. In Guangzhou, Fig 6(A) illustrates the result of the proposed IEWC is slightly superior to that of DC. However, there is no significant difference between the results of IEWC and BC (see Fig 6(B)). In Fig 6(C), it is can be seen that the curves of CC is all below the curves of IEWC and the curves of EC is the same (see Fig 6(D)). The simulations above shows that, the proposed IEWC does well in finding the key nodes of the real air route network.

**Fig 7:**
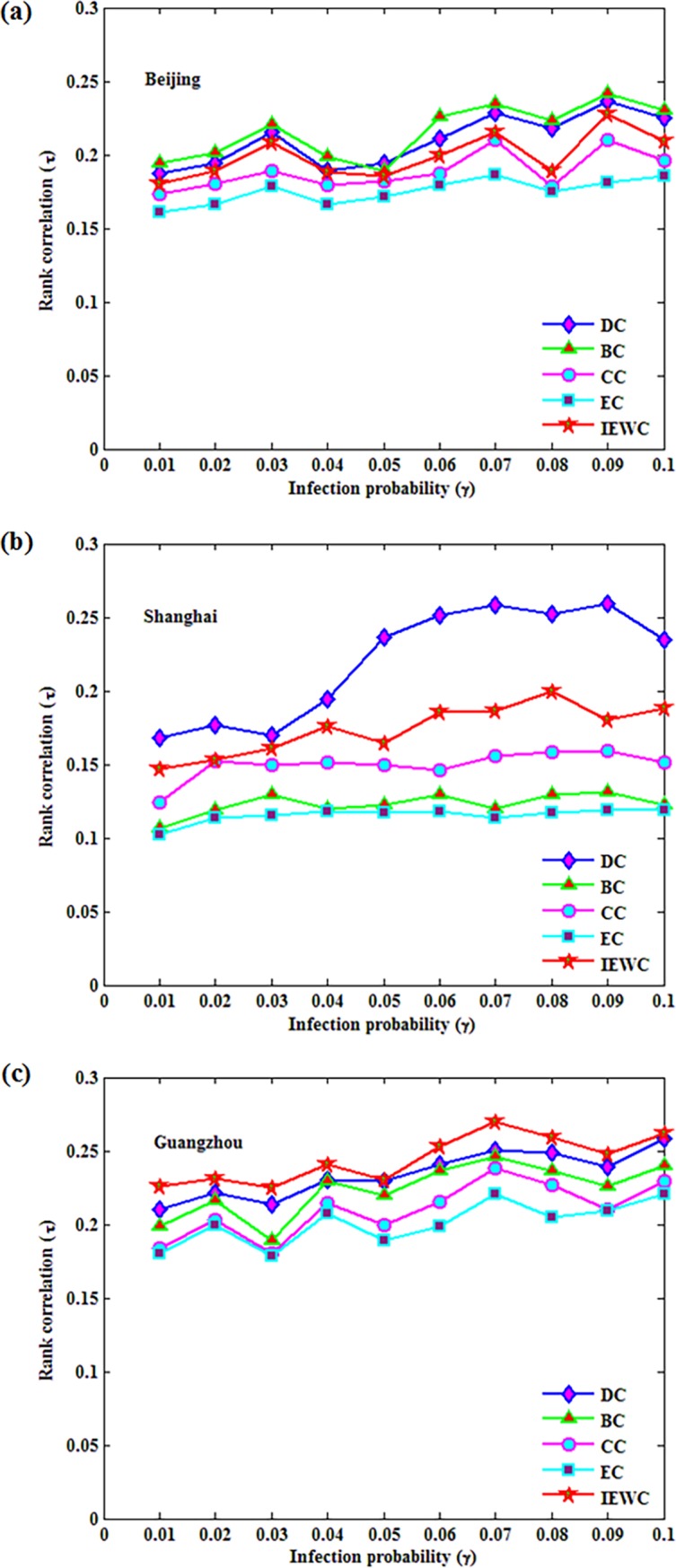
The Kendall’s tau coefficient *τ* is obtained by the infection probability *γ* varies from 0.01 to 0.1 in three air route networks. (a), (b), (c).

In addition, the correlation between the spreading ability measured by *F*(*t*) and the corresponding centrality value is analyzed by the Kendall’s tau coefficient *τ* in Fig 7. According to the theory in section 4.3, Kendall’s tau coefficient can intuitively reflect the correlation among different methods in the numerical results. In this section, the spreading probability *γ* of SIR model is gradually increasing from 0.01 to 0.1, and then the Kendall’s tau coefficient of the sorting methods and SIR model are obtained.

As shown in Fig 7(A), DC and BC perform similarly in Beijing, because the correlation curves of them are almost coincide. The correlation *τ* of the proposed IEWC is slightly lower than DC and BC, however, the performance of IEWC is relatively at upstream level in Beijing. Though CC is better than EC, but the coefficient stability is not as good as EC (see Fig 7(A)). In Shanghai, DC and the proposed IEWC do similarly when the coefficient *τ* < 0.04, meanwhile, the gap becomes larger, when *τ* > 0.04 (Fig 7(B)). The coefficient lines of BC and EC are parallel, which means that the performance of them is virtually identical in Fig 7(B). Besides, CC puts up a middle-level ranking correlation in Shanghai. From Fig 7(C), we know that the proposed method performs the best in Guangzhou, simultaneously, it is closely followed by DC. The correlation curves of BC, CC and EC in Guangzhou, are listed in the third, fourth and fifth places respectively and they are all stable (Fig 7(C)).

In brief, the top-10 key waypoints of three real air route networks ranked using the proposed method, DC, BC, CC and EC, from the results we can observe that the proposed IEWC works well. Furthermore, the performance of the proposed method does not fluctuate up and down in different air route networks. Thus, it can be seen that proposed IEWC is stable and effective.

## Conclusions

In this paper, the basic topological properties of Beijing, Shanghai and Guangzhou air route networks are analyzed by the complex network theory. The IEWC centrality method is proposed to identify influential waypoints of air route networks. The top-K nodes are ranked by the proposed method, degree centrality, betweenness centrality, closeness centrality and eigenvector centrality respectively. To evaluate the performance, the infection spreading simulations are done by the SIR model and the rank correlation between the ranking lists is compared with the Kendall’s tau coefficient. What’s more, this paper has the following research values:

1. The proposed IEWC is an objective weighting method and it extracts information from the sample, namely the original data. Furthermore, the deviation of the weight obtained by the method is smaller than that of the subjective weighting method (E.g. AHP) and the proposed method can better reflect the true importance of each evaluation index.
2. The IEWC includes not only the local (degree) and global (betweenness or closeness) characteristics of the network, but the structure features of the neighbor nodes, which can better identify the influential nodes. Besides, it has a wide range of applicability.
3. The waypoint centrality identification helps us to study the efficiency, safety and reliability of the ARN based on the existing structure. Besides, the results can provide a certain theoretical reference for the optimization and adjustment of the air routes. Thus they can they help reduce airborne delays.

The experimental results show that the proposed method can successfully identify the influential waypoints in ARNs to some degree and it has stability and effectiveness. However, the experimental data in this paper has limitations and the applicability of the theory is not well verified. In the future, we will optimize the proposed theory and extend the research to the national ARN or the world ARN. Besides, we will explore more reliable key waypoints identification methods in the context of specific aviation operations.

## Supporting information

S1 Renamed_Data The experimental air route network data in this article (csv). This document includes the adjacency matrix of the Beijing, Shanghai and Guangzhou air route networks. Renamed_46c5d (csv). The Beijing air route network. Renamed_23a33 (csv). The Shanghai air route network. Renamed_5bbbe (csv). The Guangzhou air route network.
(ZIP)
